# Performance of the LIAISON PLEX gram-positive blood culture assay for identifying bacterial pathogens and resistance genes in blood cultures

**DOI:** 10.1128/jcm.00235-26

**Published:** 2026-05-22

**Authors:** Siu-Kei Chow, Craig Stevig, Paul Granato, Neelam Dhiman, Christopher L. Emery, Rachel Behounek, Janet Farhang

**Affiliations:** 1 MultiCare Health System 14458, Tacoma, Washington, USA; 2 Laboratory Alliance of Central New York 51972 , Liverpool, New York, USA; 3 Quest Diagnostics, Lewisville, Texas, USA; 4 Indiana University Health 22529, Indianapolis, Indiana, USA; 5 Luminex Corporation 17737 , Austin, Texas, USA; Children's Hospital Los Angeles 5150, Los Angeles, California, USA

**Keywords:** gram-positive bacteria, antimicrobial resistance marker, bacteremia, sepsis, *in vitro* diagnostic assay, sample-to-answer, molecular diagnostics

## Abstract

Rapid and accurate identification of gram-positive bacteria that cause bloodstream infections using molecular assays drives antibiotic optimization and patient management. This multisite study evaluated the performance of LIAISON PLEX Gram-Positive Blood Culture (BCP) Assay, a qualitative multiplexed diagnostic molecular test that detects and differentiates 13 gram-positive bacterial targets and 4 antibiotic resistance markers (AMRs) from blood culture bottles showing gram-positive bacterial growth. Clinical performance was examined on 509 prospective, 162 pre-selected, and 225 contrived blood culture samples. The overall valid rate of the tests was 99.9%. The overall sensitivity/PPA and specificity/NPA for all targets were 98.5% and 99.7%, respectively. In the inclusivity study, 183 of 184 tested strains were detected. Cross-reactivity was observed in five bacteria among a collection of 103 off-panel species. The LIAISON PLEX BCP Assay demonstrated compatibility with various types of blood culture bottles by three manufacturers. Target organism detection was not affected by the presence of six potential interfering substances. Both sensitivity and specificity were maintained in the setting of polymicrobial infections with different organisms. The LIAISON PLEX BCP Assay allows fast detection of gram-positive organisms and their AMR genes from positive blood cultures with high accuracy and reliability.

**IMPORTANCE:**

The use of molecular assays has improved the diagnosis of bloodstream infections because of their much-reduced turnaround time and high performance compared to conventional culture workups. The early detection of clinically significant resistance markers has direct impacts on antibiotic optimization and patient isolation. This multisite study demonstrated excellent sensitivity and specificity of the LIAISON PLEX Gram-Positive Blood Culture Assay that detects and differentiates 13 gram-positive bacterial targets and 4 antibiotic resistance markers. The assay performance was assessed clinically and analytically using large and diverse sets of samples.

## INTRODUCTION

Bacterial bloodstream infection (BSI) continues to pose a significant threat to global health and is associated with high mortality and morbidity (1), also causing an economic burden (2). Gram-positive pathogens contribute to up to 50% of all BSIs (3). While the incidence of severe sepsis happens more frequently in gram-negative BSI compared to gram-positive BSI, there is no difference in survival rate or hospital stay between the two groups (4). Among BSI caused by gram-positive pathogens, *Staphylococcus aureus* is the leading bacterial cause of death in 135 countries, mostly affecting patients of >15 years old, whereas *Streptococcus pneumoniae* is associated with the most deaths in children (5). In addition to *S. aureus*, *Enterococcus faecalis* and coagulase-negative staphylococci (CoNS) are common etiologies for gram-positive BSI (6). *Staphylococcus lugdunensis* and certain *Streptococcus* species (spp.) such as *Streptococcus mutans*, *Streptococcus gallolyticus,* and *Streptococcus sanguinis* are more likely to cause infectious endocarditis (6). Over the past decade, the incidence of invasive infections caused by *Streptococcus pyogenes*, a.k.a. group A *Streptococcus*, has increased sharply (7), mostly affecting the elderly, residents of long-term care facilities, and individuals who are homeless and/or inject drugs. *Listeria* spp. remains one of the most common bacteria causing food-borne outbreaks (8). It is therefore clinically important to detect and differentiate the causative agent of gram-positive BSI on an accurate and timely basis.

Molecular diagnostics offer great performance compared to conventional microbial cultures, while significantly reducing time to identification by over 30 hours (9, 10). Most importantly, blood culture molecular panels can detect antibiotic resistance markers (AMRs), such as *mecA*, *vanA,* and *vanB*, which confer resistance to commonly used empirical antibiotics against gram-positive pathogens. Antibiotic optimization informed by molecular testing can be more than 48 hours faster than with conventional biochemical-based antimicrobial susceptibility testing (AST) methodologies. The combined advantage of reduced time to result with faster time to optimal therapy consequently shortens the length of hospital stay, facilitates patient isolation, and lowers antimicrobial cost (11, 12). In the setting of BSI caused by vancomycin-resistant *Enterococcus* (VRE), timely antibiotic escalation to anti-VRE therapy has a significant clinical benefit in 28-day mortality over empiric gram-positive coverage including vancomycin (13). Appropriate de-escalation to a beta-lactam as definitive treatment against methicillin-susceptible *S. aureus* results in 35% lower mortality compared to vancomycin (14).

This study evaluated the analytical and clinical performance of LIAISON PLEX Gram-Positive Blood Culture (BCP) Assay, a qualitative multiplexed *in vitro* diagnostic molecular test (Luminex Corporation, Austin, TX). This assay detects and differentiates 16 targets, including gram-positive bacteria commonly found to cause BSI and clinically important AMR genes associated with these bacteria.

## MATERIALS AND METHODS

### LIAISON PLEX gram-positive blood culture assay

The LIAISON PLEX BCP Assay is Food and Drug Administration (FDA)-cleared (FDA 510(k) number K243490) and detects 13 gram-positive bacteria to genus/species level, including *Bacillus*, *Listeria*, *Staphylococcus,* and *Streptococcus* at genus level; *Enterococcus faecalis*, *Enterococcus faecium*, *Staphylococcus aureus*, *Staphylococcus epidermidis*, *Staphylococcus lugdunensis*, *Streptococcus agalactiae*, *Streptococcus anginosus* group, *Streptococcus pneumoniae,* and *Streptococcus pyogenes*; and AMR genes comprising *mecA*/*mecC* (combined target), *vanA*, and *vanB*. Compared to the Verigene Gram-Positive Blood Culture Nucleic Acid Test (BC-GP), the LIAISON PLEX BCP Assay has *Bacillus* spp. and *mecC* as new targets, whereas additional or new primers were designed and employed for other targets to improve assay inclusivity and performance.

The LIAISON PLEX BCP Assay is performed on the automated LIAISON PLEX System using a single-cartridge design to test positive blood culture samples that stain gram-positive (15). Briefly, 300 µL aliquots of blood culture sample are transferred to the cartridge, followed by automated extraction of target DNA, its hybridization to complementary capture oligonucleotides, secondary hybridization by target-specific probes with gold nanoparticle conjugate, and signal enhancement. The light scatter generated is then analyzed to determine the presence (detected) or absence (not detected) of a target. Sample preparation and loading takes about 1 minute of hands-on time, and the assay run time is about 2 hours. The single-cartridge design of LIAISON PLEX BCP Assay contrasts with the multiple-component design of the Verigene BC-GP Test and also benefits from ambient-climate storage and a smaller instrument footprint.

### Samples and study design

#### Clinical performance

The study of the LIAISON PLEX BCP Assay was performed between April 2024 and September 2024 at the Luminex Clinical Operations Laboratory (Austin, TX), Luminex Molecular Diagnostics (Toronto, Ontario), and four geographically diverse clinical sites across the United States. The enrolled samples were categorized into three groups: prospective (Arm 1, *n* = 562), pre-selected (Arm 2, *n* = 163), and contrived (Arm 3, *n* = 225). Due to the projected low prevalence of certain bacterial targets in the prospective arm, additional pre-selected, de-identified remnant samples, including positives and negatives, were obtained from 10 vendors in the United States and 1 site in Europe. Clinical samples (Arm 1 and Arm 2) that met the inclusion criteria were compared to VITEK 2 (bioMérieux, Durham, NC) for bacterial isolate identification at the Luminex Clinical Operations Laboratory and to proprietary bidirectional sequencing (BDS) for AMR and *Bacillus* detection performed at Luminex Molecular Diagnostics as main comparator methodologies. The use of BDS was to supplement VITEK 2 in identifying specific targets and was performed on the Applied Biosystems 3730xl DNA Analyzer (Thermo Fisher Scientific, Waltham, MA) using the Sanger method. Primers used for BDS targeted the same genomic regions targeted by the LIAISON PLEX BCP Assay and either flanked the assay target or overlapped with the assay target. Pre-selected, remnant samples were commercially available aliquots from positive blood culture bottles, including BD BACTEC aerobic and anaerobic bottles, BacT/ALERT aerobic, anaerobic, and pediatric bottles. The aliquoted samples were shipped frozen at ≤ −70°C to the four clinical sites, followed by testing with their standard of care (SOC) methodologies of either matrix-assisted laser desorption ionization-time of flight mass spectrometry (MALDI-TOF MS) and/or molecular testing with the Verigene BC-GP and Biofire BCID2 (bioMérieux, Durham, NC) assays. To minimize bias, pre-selected specimens were tested across the same four study sites as were prospectively collected specimens, and at a central testing site (Luminex Corp.), also in a randomized, blinded manner.

For the targets that did not reach the minimum desired number of positives from the combined prospective and pre-selected groups (i.e*.*, *n* = 30 combined), additional contrived specimens (Arm 3) were tested, comprising *Bacillus* spp., *Listeria* spp., *S. lugdunensis*, and *vanB* of *Enterococcus* spp. (Table S1). To minimize bias, contrived samples were blinded, randomized, and tested along with negative clinical samples at four sites. Results from contrived samples (Arm 3) were analyzed separately from the prospective (Arm 1) and pre-selected (Arm 2) data sets. For clinical testing, all positive blood culture samples were removed from the incubator within 12 hours of bottle positivity, and then within the next 24 hours either assayed or frozen for later testing. Negative samples were cultured for at least 5 days before arriving at a negative growth determination.

#### Growth and target detection

Eleven organisms from American Type Culture Collection (ATCC) representing 16 reportable targets in the LIAISON PLEX BCP Assay were grown in BACT/ALERT FA Plus blood culture bottles (bioMérieux, Durham, NC) and evaluated by the assay at bottle positivity. In analytical performance studies, bottles were retrieved within 2 hours of initial positivity, and after an additional incubation of 8–12 hours after initial positivity. Each bottle was inoculated with 0.1 mL of a bacterial suspension in tryptic soy broth (TSB) at a 0.45–0.55 McFarland density in three bottles, and the growth was tested in triplicate, resulting in a total of nine replicates per organism. A negative blood sample was used as a negative control. Quantification was done by preparing a 6-point 10-fold dilution series in TSB of the harvested blood culture sample. The last three dilutions of the series were plated on agar plates, followed by incubation and colony counts to determine CFU/mL concentrations.

#### Inclusivity and cross-reactivity

A collection of 184 isolates, representing the genetic diversity of the analytes included in the LIAISON PLEX BCP Assay, was used to assess the inclusivity of the assay. A collection comprised of 103 off-panel species that are phylogenetically related to the on-panel organisms and likely to be present in typical blood culture samples was used to examine the cross-reactivity of the assay. Bacterial isolates were spiked into BACT/ALERT FA Plus blood culture bottles, followed by incubation and testing.

#### Interfering substances

Five endogenous substances (unconjugated bilirubin, conjugated bilirubin, hemoglobin, intralipid, and γ-globulin) and one exogenous substance (sodium polyanethol sulfonate) were used to test interference against six representative organisms (*S. aureus*, *S. epidermidis*, *S. pneumoniae*, *S. agalactiae*, *E. faecalis*, and *E. faecium*) on the LIAISON PLEX BCP Assay. Each interfering substance was tested across five replicates for each organism, in addition to the negative control without any organism. The BACT/ALERT FA Plus blood culture bottle was used in this study.

#### Media equivalency

In addition to the BACT/ALERT FA Plus blood culture bottle used in the aforementioned analytical studies (growth and detection, inclusivity and cross-reactivity, interfering substances), 12 additional types of commercially available blood culture bottles were tested for their compatibility with the LIAISON PLEX BCP Assay. A set of 17 on-panel species and 3 off-panel species (*Corynebacterium diphtheriae*, *Corynebacterium striatum*, and *Cutibacterium acnes*) were included. In this study, BACT/ALERT bottles were incubated in the BACT/ALERT 3D System, whereas the BD BACTEC bottles were incubated in BD 9050/FX40. The aerobic and anaerobic blood culture bottles by Thermo Scientific VersaTREK were incubated and monitored in a standard laboratory incubator with a shaker instead of the automated VersaTREK system.

#### Multi-site reproducibility

Site-to-site reproducibility of the LIAISON PLEX BCP Assay was evaluated by at least two operators across four sites. A reproducibility panel of 16 samples was used, which consisted of 7 representative on-panel organisms at low (initial bottle positivity) and high (initial bottle positivity +8 hours) concentrations, a negative sample spiked with *Escherichia coli* as an off-panel organism, and a negative blood culture matrix sample. All samples were blinded and randomized prior to testing. The reproducibility panel was tested in triplicate for each sample type by each operator on five non-consecutive testing days.

#### Competitive inhibition/co-infection and microbial interference

The competitive inhibition study was designed to mimic and evaluate co-infection by two on-panel pathogens, one at a lower concentration (initial bottle positivity) than the other (concentration at eight additional hours after initial bottle positivity), and vice versa. Fourteen organism-pairs were tested and checked for co-detection. The microbial interference study was conducted to mimic co-infection with potentially interfering off-panel pathogens. In a pair setting, an off-panel pathogen (*E. coli*, *Klebsiella pneumoniae*, or *Proteus mirabilis*) at a high concentration was mixed with an on-panel gram-positive pathogen (*S. epidermidis* or *E. faecium*) at a low concentration. The detection of the gram-positive pathogen was examined. In both studies, the bottles were inoculated with 0.1 mL of a bacterial suspension in TSB at a 0.45–0.55 McFarland density and grown to positivity (low concentration) or positivity plus 8 hours (high concentration). Broths containing organism pairs (high versus low concentration) were then tested with the LIAISON PLEX BCP Assay.

#### Statistical analysis

Positive and negative percent agreement (PPA and NPA) were calculated for each pathogen using the following formulae:


$$PPA=\frac{TP}{TP+FN}\times 100\%$$

$$NPA=\frac{TN}{TN+FP}\times 100\%$$

where TP, FN, TN, and FP are true positive, false negative, true negative, and false positive, respectively. Results were expressed as proportions with two-sided 95% confidence intervals (CI) according to Wilson’s score method (16). Statistical software SAS v.9.4 (SAS Corporation, Cary, NC) and Excel (Microsoft Corporation, Redmond, WA) were used.

## RESULTS

### Sample inclusion and exclusion

A total of 562 prospective and 163 pre-selected samples were enrolled in the study. Among them, 53 prospective samples and 1 pre-selected sample were excluded from the analysis. The reasons for exclusion in the prospective group included incomplete reference testing results with mixed growth when a single organism was expected, or poor or no growth of isolates (*n* = 51), duplicate patient enrollment (*n* = 1), and sample handling error (*n* = 1). The one rejected pre-selected sample was due to a sample handling error. The demographic data of the remaining, analyzed prospective (*n* = 509) and pre-selected (*n* = 162) samples are shown in Table 1. Of the 225 contrived samples, none were excluded from the analysis.

**TABLE 1:** Summary of demographics and sampling details

	Prospective (*N* = 509) #Samples (%)	Pre-selected (*N* = 162) #Samples (%)

Gender all sites		
Male	295 (58.0%)	87 (53.7%)
Female	214 (42.0%)	71 (43.8%)
Gender unknown	0 (0.0%)	4 (2.5%)
Total	509 (100.0%)	162 (100.0%)
Age (years)		
0–1	15 (2.9%)	4 (2.5%)
>1–5	0 (0.0%)	1 (0.6%)
>5–21	11 (2.2%)	5 (3.1%)
>21–65	266 (52.3%)	83 (51.2%)
>65	213 (41.8%)	57 (35.2%)
Age unknown	4 (0.8%)	12 (7.4%)
Total	509 (100.0%)	162 (100.0%)
Subject status		
Emergency room	88 (17.3%)	0 (0.0%)
Hospitalized	391 (76.8%)	4 (2.5%)
Outpatient	2 (0.4%)	0 (0.0%)
Status unknown	28 (5.5%)	158 (97.5%)
Total	509 (100.0%)	162 (100.0%)
Blood culture bottle type		
BacT/ALERT FA Plus	148 (29.1%)	48 (29.6%)
BacT/ALERT FN Plus	93 (18.3%)	31 (19.1%)
BacT/ALERT PF Plus	4 (0.8%)	2 (1.2%)
BacT/ALERT SA Standard Aerobic	27 (5.3%)	0 (0.0%)
BacT/ALERT SN Standard Anaerobic	21 (4.1%)	0 (0.0%)
BD BACTEC Lytic Anaerobic	55 (10.8%)	2 (1.2%)
BD BACTEC Peds Plus	8 (1.6%)	0 (0.0%)
BD BACTEC Plus Aerobic	135 (26.5%)	2 (1.2%)
BD BACTEC Standard Aerobic	18 (3.5%)	42 (25.9%)
BD BACTEC Standard Anaerobic	0 (0.0%)	7 (4.3%)
Bottle Type Unknown	0 (0.0%)	28 (17.3%)
Total	509 (100.0%)	162 (100.0%)

### Clinical performance

Of the 671 clinical samples from Arms 1 and 2, 646 (96.3%) generated valid results (detected or not detected) on the first attempt. One retest was allowed for each initially invalid sample due to internal control failure. Upon retest (*n* = 25), all but one sample yielded valid results, with the final success rate of LIAISON PLEX BCP Assay being 99.9%.

The clinical performance (sensitivity/PPA and specificity/NPA) of the LIAISON PLEX BCP Assay using clinical samples (Arms 1 and 2) is shown in Table 2. The overall sensitivity/PPA and specificity/NPA for all targets were 98.2% (95% CI, 97.3%–98.8%) and 99.7% (95% CI, 99.6%–99.8%), respectively. Any discrepancies between the LIAISON PLEX BCP Assay and VITEK 2/BDS were resolved by SOC methodologies (MALDI-TOF MS or Verigene BC-GP) at the four clinical sites and additional BDS for specific targets (Table 3). There was a total of 35 samples with initially discrepant results. After resolving discrepancies, the false results were reduced to 19, including 8 false positives and 11 false negatives. All result resolutions were excluded from the performance calculations per FDA requirements. Fifty-three (10.4%, 53 of 508) prospective samples were polymicrobial in the setting of: two on-panel gram-positive organisms (*n* = 3), one on-panel organism with off-panel organism(s) (*n* = 36), and mixed off-panel organisms (*n* = 14). All on-panel organisms were detected by the LIAISON PLEX BCP Assay with 100% sensitivity (data not shown).

**TABLE 2:** Clinical performance with prospective (Arm 1) and pre-selected (Arm 2) samples^*a*^

Pathogen target		Sensitivity/PPA			Specificity/NPA		
		TP / (TP + FN)	Sensitivity/PPA (%)	95% CI	TN/(TN +FP)	Specificity/NPA (%)	95% CI
**Bacteria**							
*Bacillus* spp.	Prospective	1/1	100%	20.7%–100%	506/507	99.8%	98.9%–100%
	Pre-selected	17/19	89.5%	68.6%–97.1%	127/127	100%	97.1%–100%
	**Combined**	**18/20**	**90%**	**69.9%–97.2%**	**633/634**	**99.8%**	**99.1%–100%**
*Enterococcus faecalis*	Prospective	38/38	100%	90.8%–100%	468/468	100%	99.2%–100%
	Pre-selected	2/2	100%	34.2%–100%	125/125	100%	97%–100%
	**Combined**	**40/40**	**100%**	**91.2%–100%**	**593/593**	**100%**	**99.4%–100%**
*Enterococcus faecium*	Prospective	16/16	100%	80.6%–100%	489/490	99.8%	98.9%–100%
	Pre-selected	20/20	100%	83.9%–100%	106/107	99.1%	94.9%–99.8%
	**Combined**	**36/36**	**100%**	**90.4%–100%**	**595/597**	**99.7%**	**98.8%–99.9%**
*Listeria* spp.	Prospective	0/0	N/A	N/A	506/506	100%	99.2%–100%
	Pre-selected	5/5	100%	56.6%–100%	122/122	100%	96.9%–100%
	**Combined**	**5/5**	**100%**	**56.6%–100%**	**628/628**	**100%**	**99.4%–100%**
*Staphylococcus* spp.	Prospective	316/322	98.1%	96%–99.1%	182/184	98.9%	96.1%–99.7%
	Pre-selected	20/20	100%	83.9%–100%	107/107	100%	96.5%–100%
	**Combined**	**336/342**	**98.2%**	**96.2%–99.2%**	**289/291**	**99.3%**	**97.5%–99.8%**
*Staphylococcus aureus*	Prospective	160/161	99.4%	96.6%–99.9%	344/345	99.7%	98.4%–99.9%
	Pre-selected	0/0	N/A	N/A	127/127	100%	97.1%–100%
	**Combined**	**160/161**	**99.4%**	**96.6%–99.9%**	**471/472**	**99.8%**	**98.8%–100%**
*Staphylococcus epidermidis*	Prospective	93/96	96.9%	91.2%–98.9%	405/410	98.8%	97.2%–99.5%
	Pre-selected	0/0	N/A	N/A	126/127	99.2%	95.7%–99.9%
	**Combined**	**93/96**	**96.9%**	**91.2%–98.9%**	**531/537**	**98.9%**	**97.6%–99.5%**
*Staphylococcus lugdunensis*	Prospective	6/6	100%	61%–100%	500/500	100%	99.2%–100%
	Pre-selected	20/20	100%	83.9%–100%	107/107	100%	96.5%–100%
	**Combined**	**26/26**	**100%**	**87.1%–100%**	**607/607**	**100%**	**99.4%–100%**
*Streptococcus* spp.	Prospective	97/98	99%	94.4%–99.8%	406/408	99.5%	98.2%–99.9%
	Pre-selected	78/80	97.5%	91.3%–99.3%	47/47	100%	92.4%–100%
	**Combined**	**175/178**	**98.3%**	**95.2%–99.4%**	**453/455**	**99.6%**	**98.4%–99.9%**
*Streptococcus agalactiae*	Prospective	21/21	100%	84.5%–100%	485/485	100%	99.2%–100%
	Pre-selected	17/17	100%	81.6%–100%	110/110	100%	96.6%–100%
	**Combined**	**38/38**	**100%**	**90.8%–100%**	**595/595**	**100%**	**99.4%–100%**
*Streptococcus anginosus* group	Prospective	8/9	88.9%	56.5%–98%	496/497	99.8%	98.9%–100%
	Pre-selected	25/26	96.2%	81.1%–99.3%	101/101	100%	96.3%–100%
	**Combined**	**33/35**	**94.3%**	**81.4%–98.4%**	**597/598**	**99.8%**	**99.1%–100%**
*Streptococcus pneumoniae*	Prospective	11/11	100%	74.1%–100%	494/495	99.8%	98.9%–100%
	Pre-selected	21/21	100%	84.5%–100%	106/106	100%	96.5%–100%
	**Combined**	**32/32**	**100%**	**89.3%–100%**	**600/601**	**99.8%**	**99.1%–100%**
*Streptococcus pyogenes*	Prospective	21/21	100%	84.5%–100%	485/485	100%	99.2%–100%
	Pre-selected	16/16	100%	80.6%–100%	111/111	100%	96.7%–100%
	**Combined**	**37/37**	**100%**	**90.6%–100%**	**596/596**	**100%**	**99.4%–100%**
Resistance Marker Genes							
*mecA/mecC*	Prospective	155/157	98.7%	95.5%–99.6%	154/161	95.7%	91.3%–97.9%
	Pre-selected	3/3	100%	43.9%–100%	18/18	100%	82.4%–100%
	**Combined**	**158/160**	**98.8%**	**95.6%–99.7%**	**172/179**	**96.1%**	**92.1%–98.1%**
*vanA*	Prospective	8/8	100%	67.6%–100%	47/47	100%	92.4%–100%
	Pre-selected	22/22	100%	85.1%–100%	1/1	100%	20.7%–100%
	**Combined**	**30/30**	**100%**	**88.6%–100%**	**48/48**	**100%**	**92.6%–100%**
*vanB*	Prospective	1/1	100%	20.7%–100%	54/54	100%	93.4%–100%
	Pre-selected	0/0	N/A	N/A	23/23	100%	85.7%–100%
	**Combined**	**1/1**	**100%**	**20.7%–100%**	**77/77**	**100%**	**95.2%–100%**

^*a*^ The bold values are combined performance of prospective and pre-selected.

**TABLE 3:** Samples with discordant BCP results and corresponding SOC results^*e*^

Sample information		Testing results (Included in performance)				Standard of care		
Sample	Cohort	LIAISON PLEX BCP result	VITEK 2 result	Interpretation	Discordant target(s)	SOC molecular result	SOC MALDI-TOF result	Resolved in agreement with BCP
1	Prospective	*Bacillus* spp.	*Staphylococcus vitulinus*	FP | FN	*Bacillus* spp. (FP) *Staphylococcus* spp. (FN)	NA	*Bacillus* spp*.*	Y
2	Prospective	*Enterococcus faecium*	*Enterococcus avium*	FP	*Enterococcus faecium*	*Enterococcus faecium*	NA	Y
3	Pre-selected	*Enterococcus faecium Staphylococcus epidermidis Staphylococcus lugdunensis Staphylococcus spp.*	*Staphylococcus lugdunensis*	FP	*Enterococcus faecium Staphylococcus epidermidis*	*Staphylococcus lugdunensis*	NA	N
4	Prospective	None	*Staphylococcus auricularis*	FN	*Staphylococcus* spp*.*	*Staphylococcus* spp*.*	NA	N
5	Prospective	None	*Staphylococcus hominis* ssp. *hominis Staphylococcus warneri*	FN	*Staphylococcus* spp*.*	*Staphylococcus* spp*.*	NA	N
6	Prospective	None	*Staphylococcus hominis* ssp. *hominis*	FN	*Staphylococcus* spp.^*a*^	*Staphylococcus* spp*.*, mecA/C	NA	N
7	Prospective	None	*Staphylococcus epidermidis*	FN	*Staphylococcus* spp*. Staphylococcus epidermidis*	Negative for *S. epidermidis*	*Bacillus* species, not *B. anthracis*	Y
8	Prospective	None	*Staphylococcus saccharolyticus*	FN	*Staphylococcus* spp*.*	*Staphylococcus* spp*.*	*Staphylococcus saccharolyticus*	N
9	Prospective	*Staphylococcus epidermidis Staphylococcus* spp*.* mecA/mecC	*Rothia kristinae*	FP	*Staphylococcus* spp*. Staphylococcus epidermidis* mec A/C	*Staphylococcus epidermidis*, mecA/C	NA	Y
10	Prospective	*Staphylococcus* spp*.* mecA/mecC	*Aerococcus viridans*	FP	*Staphylococcus* spp. mec A/C	*Staphylococcus* spp*.*, mecA/C	*Staphylococcus hominis*	Y
11	Prospective	*Staphylococcus aureus Staphylococcus* spp*.*	*Staphylococcus pseudintermedius*	FP	*Staphylococcus aureus*	*Staphylococcus aureus*	NA	Y
12	Prospective	*Staphylococcus* spp. mecA/mecC	*Staphylococcus epidermidis*	FN	*Staphylococcus epidermidis*	*Staphylococcus epidermidis*, mecA/C	NA	N
13	Prospective	*Staphylococcus* spp.	*Staphylococcus epidermidis*	FN	*Staphylococcus epidermidis* mecA/C	NA^*d*^	*Staphylococcus epidermidis*	N
14	Prospective	*Staphylococcus epidermidis Staphylococcus* spp*.* mecA/mecC	*Staphylococcus hominis* ssp. *hominis*	FP	*Staphylococcus epidermidis*	*Staphylococcus epidermidis*, mecA/C	*Staphylococcus epidermidis*	Y
15	Prospective	*Staphylococcus epidermidis Staphylococcus* spp*.* mecA/mecC	*Staphylococcus haemolyticus*	FP	*Staphylococcus epidermidis*	*Staphylococcus* spp.^*d*^	*Staphylococcus epidermidis*	Y
16	Prospective	*Staphylococcus epidermidis Staphylococcus* spp*.* mecA/mecC	*Staphylococcus capitis*	FP	*Staphylococcus epidermidis*	*Staphylococcus epidermidis*, mecA/C	*Staphylococcus epidermidis*	Y
17	Prospective	*Staphylococcus epidermidis Staphylococcus* spp*.* mecA/mecC	*Staphylococcus hominis* ssp. *hominis*	FP	*Staphylococcus epidermidis*	*Staphylococcus epidermidis* ^*d*^	*Staphylococcus epidermidis*	Y
18	Prospective	*Streptococcus* spp*.*	*Lactococcus lactis* ssp. *lactis*	FP	*Streptococcus* spp*.*	*Streptococcus* spp*.*	*Lactococcus lactis*	Y
19	Prospective	*Streptococcus* spp*.*	*Kocuria rosea Granulicatella adiacens*	FP	*Streptococcus* spp*.*	Streptococcus spp.	*Streptococcus mitis/Streptococcus oralis*	Y
20	Prospective	*Streptococcus anginosus* group *Streptococcus* spp*.*	*Streptococcus gordonii*	FP	*Streptococcus anginosus* group	*Streptococcus anginosus* group	NA	Y
21	Pre-selected	None	NA	FN	*Bacillus* spp.^*b*^	NA	NA	U
22	Prospective	*Staphylococcus* spp*.* mecA/mecC	*Staphylococcus aureus*	FN	*Staphylococcus aureus*	*Staphylococcus aureus* ^*d*^	*Staphylococcus aureus*	N
23	Prospective	None	*Streptococcus intermedius*	FN	*Streptococcus* spp*. Streptococcus anginosus* group	*Streptococcus* spp*.*	*Streptococcus* spp*.*	N
24	Pre-selected	None	*Streptococcus anginosus*	FN	*Streptococcus* spp*. Streptococcus anginosus* group	*Streptococcus anginosus*	NA	N
25	Pre-selected	*Streptococcus anginosus* group	*Streptococcus anginosus*	FN	*Streptococcus* spp*.*	*Streptococcus anginosus*	NA	N
26	Prospective	*Streptococcus pneumoniae Streptococcus* spp*.*	*Streptococcus mitis/Streptococcus oralis*	FP	*Streptococcus pneumoniae*	*Staphylococcus epidermidis*, mecA/C	*Streptococcus mitis*	N
27	Prospective	*Staphylococcus epidermidis Staphylococcus* spp*.*	*Staphylococcus epidermidis*	FN	mecA/C	*Staphylococcus epidermidis* ^*d*^	*Staphylococcus epidermidis*	N
28	Prospective	*Staphylococcus spp.* mecA/mecC	*Staphylococcus hominis* ssp. *hominis*	FP	mecA/C	*Staphylococcus spp.*	NA	N
29	Prospective	*Staphylococcus epidermidis Staphylococcus* spp*.* mecA/mecC	*Staphylococcus epidermidis*	FP	mecA/C^*c*^	NA	*Staphylococcus epidermidis*	U
30	Prospective	*Staphylococcus* spp. mecA/mecC	*Staphylococcus capitis Staphylococcus hominis* ssp. *hominis staphylococcus warneri*	FP	mecA/C	*Staphylococcus spp.*	*Staphylococcus hominis*	N
31	Prospective	*Staphylococcus* spp*.* mecA/mecC	*Staphylococcus capitis Staphylococcus hominis* ssp. *hominis Staphylococcus saprophyticus*	FP	mecA/C	*Staphylococcus* spp*.*	*Staphylococcus hominis | Staphylococcus hominis*	N
32	Prospective	*Staphylococcus epidermidis Staphylococcus* spp*.* mecA/mecC	*Staphylococcus epidermidis*	FP	mecA/C	*Staphylococcus epidermidis*, mecA/C	*Staphylococcus epidermidis*	Y
33	Prospective	*Staphylococcus epidermidis Staphylococcus* spp*.* mecA/mecC	*Staphylococcus epidermidis*	FP	mecA/C	*Staphylococcus lugdunensis*	*Staphylococcus epidermidis*	N
34	Prospective	*Staphylococcus epidermidis Staphylococcus* spp*. Streptococcus* spp*.* mecA/mecC	N/A (Isolate 1) | *Staphylococcus epidermidis* (Isolate 2) | *Streptococcus mitis/Streptococcus oralis* | *Streptococcus pneumoniae* (Isolate 3)	FP	mecA/C	*Staphylococcus epidermidis, Streptococcus* spp*.*	*Clostridium perfringens Staphylococcus epidermidis Streptococcus mitis/oralis*	N
35	Pre-selected	*Bacillus* spp*.*	NA	FP	*Bacillus* spp*.*	*Corynebacterium striatum*	NA	N

^*a*^ *mecA*/*C* discrepancy not counted because primary *Staphylococcus* genus target not detected.

^*b*^ Pre-selected *Bacillus*-positive sample, but SOC results not available.

^*c*^ *mecA*/*C* PCR result from the gold standard is not available.

^*d*^ BDS result was positive for *mecA/mecC*.

^*e*^ Abbreviations used: SOC, standard of care; NA, not available; FP, false positive; FN, false negative; spp., species.

The contrived sample study examined low-prevalence targets: *Bacillus* spp., *Listeria* spp., *S. lugdunensis*, and *vanB* of *Enterococcus* spp. (Table S1). Of the 225 contrived samples, 211 samples (93.8%) generated valid results on the first attempt. Upon retesting the remaining 14 samples once, the final testing success rate for all samples reached 100%. Both sensitivity/PPA and specificity/NPA were 100% (Table S2).

The LIAISON PLEX BCP Assay reports genus-level results for *Bacillus* spp., *Staphylococcus* spp., and *Streptococcus* spp. The sensitivity/PPA for each of these target organisms stratified at the species level is shown in Table S3. The samples with reference results of low discrimination between two species of the same genus that were not further resolved were listed as unknown species. The clinical performance for each resistance marker stratified by eligible organisms is listed in Table S4 through S6.

### Analytical performance

In the growth and target detection study, all tested samples spiked with 11 individual organisms spanning a total of 16 LIAISON PLEX BCP Assay targets demonstrated 100% positivity (Table S7). The average concentrations of the organisms at initial bottle positivity ranged from 1.52 × 10^7^ to 1.32 × 10^9^ CFU/mL, and after an additional incubation of 8 hours, ranged from 2.90 × 10^5^ to 2.90 × 10^9^ CFU/mL.

The inclusivity of the LIAISON PLEX BCP Assay was examined using 184 isolates representing the genetic diversity of the analytes on the assay, which revealed positivity of 100% for all tested species except for *Streptococcus mutans* (Table S8). Cross-reactivity was observed between *Staphylococcus argenteus* and *S. aureus*, and *Staphylococcus muscae* and *Listeria* spp. A detection rate of 100% was observed in the 56 tested strains of *Staphylococcus* and *Enterococcus* spp. harboring *mecA/mecC*, *vanA*, or *vanB* (data not shown). Cross-reactivity of the LIAISON PLEX BCP Assay was studied with 103 off-panel, closely related species, 5 of which demonstrated cross-reaction. *Enterococcus avium* was detected as *E. faecium; Leuconostoc carnosum*, *Aggregatibacter aphrophilus*, *Proteus penneri,* and *Proteus vulgaris* were detected as *Streptococcus* spp. These cross-reactivities were predicted by *in silico* analysis of the LIAISON PLEX BCP Assay oligo designs (data not shown).

In the interfering substances study, 100% target detection was observed in the presence and absence of all six interfering substances (Table S9). The use of 12 additional types of blood culture bottles for the LIAISON PLEX BCP Assay generated expected results in both spiked positive samples and negative control matrix bottles (Table 4). The list of 17 on-panel species and 3 off-panel species with the range of concentrations across all bottle types is shown in Table S10. The multi-site reproducibility study revealed 99.7% reproducibility (2,783 of 2,790 matched results) of the LIAISON PLEX BCP Assay (data not shown).

**TABLE 4:** Media equivalency study showing assay performance with 12 blood culture bottles

Manufacturer System	Blood Culture Bottle Manufacturer	Blood Culture Bottle Type		Unique bottles		
				Seeded Organisms Negative Blood Matrix		
				Gram-positive bacteria	Gram-negative bacteria	Negative blood matrix^*c*^
					Expected results	
bioMérieux BACT/ALERT 3D System	bioMérieux BACT/ALERT	Aerobic	BACT/ALERT SA	20/20	10/10	1/1
		Anaerobic	BACT/ALERT SN	20/20	10/10	1/1
			BACT/ALERT FN Plus	20/20	10/10	1/1
		Pediatric	BACT/ALERT PF Plus	58/58	10/10	1/1
Becton Dickinson 9050/FX40	Becton Dickinson BACTEC	Aerobic	BACTEC Standard	20/20^*b*^	10/10	1/1
			BACTEC Plus	20/20	10/10	1/1
		Anaerobic	BACTEC Standard	58/58^*b*^	10/10	1/1
			BACTEC Plus	58/58	10/10	1/1
		Pediatric	BACTEC Peds Plus	58/58	10/10	1/1
		Lytic Anaerobic	BACTEC Lytic	20/20	10/10	1/1
N/A^*a*^	Thermo Scientific VersaTREK	Aerobic	REDOX 1 EZ Draw	56/56	10/10	1/1
		Anaerobic	REDOX 2 EZ Draw	56/56	10/10	1/1
Bottles with expected results/total number blood bottles				464/464^*b*^(100%)	120/120 (100%)	12/12 (100%)

^*a*^ The aerobic and anaerobic blood culture bottles by Thermo Scientific VersaTREK were incubated and monitored in a standard laboratory incubator with a shaker instead of the automated VersaTREK system. BACT/ALERT bottles were incubated in a BACT/ALERT 3D System, and BD BACTEC bottles were incubated in a BD 9050/FX40 system.

^*b*^ One organism, (*E. faecalis* strain 51575) required use of high concentration (initial bottle positivity +8 hours) bottles to confirm the bottle matrix was not inhibitory.

^*c*^ Each unique bottle of negative blood matrix was tested in replicates of three.

The competitive inhibition study was performed in 14 organism-pairs to examine co-detection of any 2 given on-panel pathogens at different concentrations, which revealed 100% co-detection (Table S11). Similarly, in a pair setting, an off-panel pathogen at a high concentration was mixed with an on-panel pathogen at a low concentration, and no microbial interference was detected (Table S12).

## DISCUSSION

This study revealed high concordance between the LIAISON PLEX BCP Assay and its comparator testing methods in detecting and differentiating gram-positive organisms that are common causes of BSI. Among the 16 assay targets, *Bacillus* spp. and *mecC* were new targets compared to the Verigene Gram-Positive Blood Culture Test. The addition of the *Bacillus* target allows rapid detection of BSI caused by this group of opportunistic pathogens that occur particularly in the setting of catheter-related BSI (17). When *Bacillus* causes blood culture contamination due to suboptimal skin preparation prior to blood draw, the use of the LIAISON PLEX BCP Assay in conjunction with clinical examination may raise attention to potential contamination and reduce unnecessary antibiotic use. The BCP Assay’s *Bacillus* spp. target is purposefully designed to be inclusive of the *B. cereus* group (25 of 29 species) and the *B. subtilis* group (15 of 15 species). While *mecC* is more common in veterinary cases, *mecC*-methicillin-resistant *S. aureus* (*mecC*-MRSA) human infections have been reported for more than a decade, with an overall prevalence of 2.41% (18).

The performance of a nucleic acid-based assay depends on the nature of the test and its probe design. Molecular hybridization compared to amplification-based PCR test is less likely to generate false-positive results from the DNA of non-viable organisms or other contaminated DNA in blood culture media (19). The LIAISON PLEX BCP Assay demonstrated an excellent sensitivity/PPA of 98.2% and specificity/NPA of 99.7% in the clinical arms. While the Verigene BC-GP was the predecessor of LIAISON PLEX BCP Assay, the latest assay contains several new targets and updated oligo sets for 8 of 15 shared targets, overall increasing BCP Assay inclusivity and sensitivity (not shown). In previous studies of the Verigene BC-GP Test, false-positive results were observed such that certain cultures with *S. mitis/S. oralis* or *S. anginosus* group were misidentified as *S. pneumoniae* (20, 21). In the three clinical arms of the current study, only one *S*. *mitis* was misidentified as *S. pneumoniae*. In the inclusivity study, additional testing of *S. mitis* (*n* = 2 strains), *S. oralis* (*n* = 2), and *S. anginosus* group (*n* = 7) did not show any false detection of *S. pneumoniae*. In our study, BDS was used as the primary comparator for AMR genes and *Bacillus* species. MALDI-TOF MS cannot identify AMR genes using typical approaches and has known limitations in identifying *Bacillus* with standard databases (22, 23).

This study has several limitations. First, the performance of the LIAISON PLEX BCP Assay applied to the tested strains and may deviate due to strain differences in the actual clinical practice. Second, given that most BSIs are caused by a small subset of organisms among the targets of the panel, the statistical power for organisms with low prevalence was lower. While contrived samples of reference strains may be limited in clinical relevance, they helped supplement the study when the total number of prospective and pre-selected clinical samples was lower than the desired number of at least 30 strains per target. Third, in the media equivalency study, the VersaTREK blood culture bottles were incubated and monitored in a standard laboratory incubator with a shaker instead of the automated VersaTREK system. The potential variation in growth patterns of tested bacteria was not examined, although the performance matched the expected results. Lastly, some inconsistency may have resulted from the use of different SOC comparator methods across clinical study sites.

As predicted by the *in silico* analysis of the LIAISON PLEX BCP Assay oligonucleotide designs and subsequently confirmed in the inclusivity study, *S. argenteus,* a member of *S. aureus* complex, cross-reacted with the *S. aureus* target. *Staphylococcus muscae* was falsely detected as *Listeria* spp., but the difference in gram-stain morphologies should alert the laboratory technician of this misidentification. In the cross-reactivity study, *E. avium* was shown to cross-react with the *E. faecium* target on the assay, whereas *L. carnosum* cross-reacted with the *Streptococcus* spp. target. Other cross-reactivities between the *Streptococcus* spp. assay target and gram-negative organisms, that is, *A. aphrophilus*, *P. penneri,* and *P. vulgaris*, can all be resolved by gram staining prior to running the LIAISON PLEX BCP Assay.

The use of rapid molecular panels (RMPs) to help diagnose BSI and optimize antibiotic treatment has been widely adopted in the fields of clinical microbiology, infectious diseases, and infection prevention. From a recent meta-analysis study, the combined use of RMPs and an antimicrobial stewardship program (ASP) resulted in a significant reduction in mortality compared to conventional blood culture with or without ASP (24). Most importantly, RMPs alone without ASP did not show benefit in survival. A reduction in hospital length of stay was observed in the group of RMP and ASP compared to that of conventional blood culture. A reduced time to optimal therapy relies strictly on both RMP and ASP. The hospital and the laboratory must have a plan or algorithm to act on the positive results of the RMPs, especially when it is related to the detection of multidrug-resistant organisms. Efficient consultation with infectious disease providers improves patient management and reduces mortality (25, 26). Continuous work on standardizing the best practice in fast diagnostics, antibiotic de-escalation, and patient management is the key to reduce in-hospital mortality and sepsis-mediated sequelae (27, 28). The education and awareness of minimizing blood culture contamination will facilitate the use of RMP to improve its clinical utility in a cost-effective manner.

The LIAISON PLEX BCP Assay rapidly interrogates positive blood cultures for the presence of a broad panel of gram-positive bacterial pathogens commonly responsible for BSI and their AMR genes. Given the demonstrated high sensitivity and specificity of the BCP assay, it deserves consideration for use as an RMP to supplement other clinical and laboratory findings in diagnosing the causative organism in gram-positive BSI.

## Data Availability

The data supporting the findings of the study are available from the corresponding author upon reasonable request.

## SUPPLEMENTAL MATERIAL

Supplemental tables jcm.00235-26-s0001.pdf Tables S1 to S12.

ASM does not own the copyrights to Supplemental Material that may be linked to, or accessed through, an article. The authors have granted ASM a non-exclusive, world-wide license to publish the Supplemental Material files. Please contact the corresponding author directly for reuse.
