# Supplementary material for: Performance of the LIAISON PLEX gram-positive blood culture assay for identifying bacterial pathogens and resistance genes in blood cultures
Source: J Clin Microbiol. 2026 May 22;64(6):e00235-26. doi: 10.1128/jcm.00235-26 (PMC13251389; doi:10.1128/jcm.00235-26)
Supplement: Supplemental tables — Tables S1 to S12. [file jcm.00235-26-s0001.pdf]

Table S1: Summary of contrived samples

Organism	Resistance Marker(s)	Strain	# of Independent Samples Tested
Bacillus amyloliquefaciens	N/A	ATCC 23350	10
Bacillus atrophaeus	N/A	ATCC 6455	10
Bacillus cereus	N/A	ATCC 11778	10
Bacillus licheniformis	N/A	ATCC 14580	10
Bacillus thuringiensis	N/A	ATCC 10792	10
***Bacillus* spp. Total**			**50**
Enterococcus faecalis	*vanB*	ATCC 700802	10
		ATCC 51299	10
		64188262	10
***Enterococcus faecalis* Total**			**30**
Enterococcus faecium	*vanB*	ATCC 51858	10
		JMI CS-712	10
***Enterococcus faecium* Total**			**20**
Listeria grayi	N/A	ATCC 25401	10
Listeria innocua	N/A	ATCC 33090	10
Listeria ivanovii	N/A	ATCC 19119	10
Listeria monocytogenes	N/A	ATCC 19114	8
Listeria welshimeri	N/A	ATCC 35897	11
***Listeria* spp. Total**			**49**
Staphylococcus lugdunensis	N/A	ATCC 84497462	50
***Staphylococcus lugdunensis* Total**			**50**

Table S2: Sensitivity/PPA and Specificity/NPA of contrived samples (Arm 3)

Pathogen Target	Sensitivity/PPA			Specificity/NPA		
**Analyte**	**TP / (TP+FN)**	**Sensitivity/PPA (%)**	**95% CI**	**TN / (TN+FP)**	**Specificity/NPA (%)**	**95% CI**
**Bacteria**						
Bacillus spp.	50/50	100%	92.9%–100%	175/175	100%	97.9%–100%
Enterococcus faecalis	30/30	100%	88.6%–100%	195/195	100%	98.1%–100%
Enterococcus faecium	20/20	100%	83.9%–100%	205/205	100%	98.2%–100%
Listeria spp.	49/49	100%	92.7%–100%	176/176	100%	97.9%–100%
Staphylococcus spp.	50/50	100%	92.9%–100%	175/175	100%	97.9%–100%
Staphylococcus aureus	0/0	N/A	N/A	225/225	100%	98.3%–100%
Staphylococcus epidermidis	0/0	N/A	N/A	225/225	100%	98.3%–100%
Staphylococcus lugdunensis	50/50	100%	92.9%–100%	175/175	100%	97.9%–100%
Streptococcus spp.	0/0	N/A	N/A	225/225	100%	98.3%–100%
Streptococcus agalactiae	0/0	N/A	N/A	225/225	100%	98.3%–100%
Streptococcus anginosus group	0/0	N/A	N/A	225/225	100%	98.3%–100%
Streptococcus pneumoniae	0/0	N/A	N/A	225/225	100%	98.3%–100%
Streptococcus pyogenes	0/0	N/A	N/A	225/225	100%	98.3%–100%
**Resistance Marker Genes[a]**						
mecA/mecC	0/0	N/A	N/A	50/50	100%	92.9%–100%
vanA	0/0	N/A	N/A	50/50	100%	92.9%–100%
vanB	50/50	100%	92.9%–100%	0/0	N/A	N/A

[a]The LIAISON PLEX® BCP Assay will report the presence or absence of resistance markers only if an applicable organism is also detected.

Table S3: Sensitivity/PPA of genus-level targets, stratified by species

Organism	Prospective (Arm 1)		Pre-selected (Arm 2)		Contrived (Arm 3)	
	Sensitivity/PPA	95% CI	Sensitivity/PPA	95% CI	Sensitivity/PPA	95% CI
Bacillus spp.	100% (1/1)	20.7%–100%	89.5% (17/19)	68.6%–97.1%	100% (50/50)	92.9%–100%
Bacillus amyloliquefaciens	N/A	N/A	N/A	N/A	100% (10/10)	72.2%–100%
Bacillus atrophaeus	N/A	N/A	N/A	N/A	100% (10/10)	72.2%–100%
Bacillus cereus	N/A	N/A	N/A	N/A	100% (10/10)	72.2%–100%
Bacillus licheniformis	N/A	N/A	N/A	N/A	100% (10/10)	72.2%–100%
Bacillus thuringiensis	N/A	N/A	N/A	N/A	100% (10/10)	72.2%–100%
Listeria spp.	NA	NA	100% (5/5)	56.6%–100%	100% (49/49)	92.7%–100%
Listeria grayi	N/A	N/A	N/A	N/A	100% (10/10)	72.2%–100%
Listeria innocua	N/A	N/A	N/A	N/A	100% (10/10)	72.2%–100%
Listeria ivanovii	N/A	N/A	N/A	N/A	100% (10/10)	72.2%–100%
Listeria monocytogenes	N/A	N/A	100% (5/5)	56.6%–100%	100% (8/8)	67.6%–100%
Listeria welshimeri	N/A	N/A	N/A	N/A	100% (11/11)	74.1%–100%
Staphylococcus spp.	98.1% (316/322)	96%–99.1%	100% (20/20)	83.9%–100%	100% (50/50)	92.9%–100%
Staphylococcus aureus	99.4% (160/161)	96.6%–99.9%	N/A	N/A	N/A	N/A
Staphylococcus epidermidis	96.9% (93/96)	91.2%–98.9%	N/A	N/A	N/A	N/A
Staphylococcus lugdunensis	100% (6/6)	61%–100%	100% (20/20)	83.9%–100%	100% (50/50)	92.9%–100%
Staphylococcus arlettae	100% (1/1)	20.7%–100%	N/A	N/A	N/A	N/A
Staphylococcus auricularis	66.7% (2/3)	20.8%–93.9%	N/A	N/A	N/A	N/A
Staphylococcus capitis	100% (9/9)	70.1%–100%	N/A	N/A	N/A	N/A
Staphylococcus caprae	100% (1/1)	20.7%–100%	N/A	N/A	N/A	N/A
Staphylococcus haemolyticus	100% (6/6)	61%–100%	N/A	N/A	N/A	N/A
Staphylococcus hominis	96.3% (31/32)	84.3%–99.4%	N/A	N/A	N/A	N/A
Staphylococcus pseudintermedius	100% (1/1)	20.7%–100%	N/A	N/A	N/A	N/A
Staphylococcus saccharolyticus	0% (0/1)	0%–79.3%	N/A	N/A	N/A	N/A
Staphylococcus saprophyticus	100% (1/1)	20.7%–100%	N/A	N/A	N/A	N/A
Staphylococcus simulans	100% (1/1)	20.7%–100%	N/A	N/A	N/A	N/A
Staphylococcus vitulinus	0% (0/1)	0%–79.3%	N/A	N/A	N/A	N/A
Staphylococcus warneri	100% (3/3)	43.9%–100%	N/A	N/A	N/A	N/A

Staphylococcus spp. Unknown	87.5% (7/8)	52.9%–97.8%	N/A	N/A	N/A	N/A
Streptococcus spp.	99% (97/98)	94.4%–99.8%	97.5% (78/80)[a]	91.3%–99.3%	N/A	N/A
Streptococcus agalactiae	100% (21/21)	84.5%–100%	100% (17/17)	81.6%–100%	N/A	N/A
Streptococcus anginosus	88.9% (8/9)	56.5%–98%	96.2% (25/26)	81.1%–99.3%	N/A	N/A
Streptococcus pneumoniae	100% (11/11)	74.1%–100%	100% (21/21)	84.5%–100%	N/A	N/A
Streptococcus pyogenes	100% (21/21)	84.5%–100%	100% (16/16)	80.6%–100%	N/A	N/A
Streptococcus dysgalactiae	100% (15/15)	79.6%–100%	N/A	N/A	N/A	N/A
Streptococcus gallolyticus	100% (1/1)	20.7%–100%	N/A	N/A	N/A	N/A
Streptococcus gordonii	100% (4/4)	51%–100%	N/A	N/A	N/A	N/A
Streptococcus parasanguinis	100% (1/1)	20.7%–100%	N/A	N/A	N/A	N/A
Streptococcus salivarius	100% (2/2)	34.2%–100%	N/A	N/A	N/A	N/A
Streptococcus sanguinis	100% (2/2)	34.2%–100%	N/A	N/A	N/A	N/A
Streptococcus spp. Unknown	100% (11/11)	74.1%–100%	N/A	N/A	N/A	N/A

[a]For one sample the assay returned a true positive call for *Streptococcus anginosus* without detecting *Streptococcus* spp. As a result, there are two total FNs for the *Streptococcus* spp. target, but only one listed when stratified by species.

Table S4: Performance of *mecA/mecC*, stratified by organism

Organism	Prospective (Arm 1)		Pre-selected (Arm 2)		Contrived (Arm 3)	
	Sensitivity/PPA	95% CI	Sensitivity/PPA	95% CI	Sensitivity/PPA	95% CI
Staphylococcus aureus	100% (64/64)	94.3%–100%	N/A	N/A	N/A	N/A
Staphylococcus epidermidis	97% (65/67)	89.8%–99.2%	N/A	N/A	N/A	N/A
Staphylococcus lugdunensis	100% (4/4)	51%–100%	100% (3/3)	43.8%–100%	N/A	N/A
Staphylococcus capitis	100% (2/2)	34.2%–100%	N/A	N/A	N/A	N/A
Staphylococcus haemolyticus	100% (6/6)	61.0%–100%	N/A	N/A	N/A	N/A
Staphylococcus hominis	100% (12/12)	75.7%–100%	N/A	N/A	N/A	N/A
Spp. unknown	100% (2/2)	34.2%–100%	N/A	N/A	N/A	N/A
**Overall**	98.70% (155/157)	95.4%–99.6%	100% (3/3)	43.8%–100%	N/A	N/A

Table S5: Performance of *vanA*, stratified by organism

Organism	Prospective (Arm 1)		Pre-selected (Arm 2)		Contrived (Arm 3)	
	Sensitivity/PPA	95% CI	Sensitivity/PPA	95% CI	Sensitivity/PPA	95% CI
Enterococcus faecalis	N/A	N/A	100% (2/2)	34.2%–100%	N/A	N/A
Enterococcus faecium	100% (8/8)	67.6%–100%	100% (20/20)	83.9%–100%	N/A	N/A
**Overall**	100% (8/8)	67.6%–100%	100% (22/22)	85.1%–100%	N/A	N/A

Table S6: Performance of *vanB*, stratified by organism

Organism	Prospective (Arm 1)		Pre-selected (Arm 2)		Contrived (Arm 3)	
	Sensitivity/PPA	95% CI	Sensitivity/PPA	95% CI	Sensitivity/PPA	95% CI
Enterococcus faecalis	N/A	N/A	N/A	N/A	100% (30/30)	88.6%–100%
Enterococcus faecium	100% (1/1)	20.7%–100%	N/A	N/A	100% (20/20)	83.9%–100%
**Overall**	100% (1/1)	20.7%–100%	N/A	N/A	100% (50/50)	92.9%–100%

Table S7: Growth and detection study summary

Organism Tested	Expected Targets	Initial Bottle Positivity		8 Hours After Initial Bottle Positivity	
		Per Bottle (CFU/mL)	Positive Agreement/ Total (% Detected)	Per Bottle (CFU/mL)	Positive Agreement/ Total (% Detected)
Bacillus subtilis ATCC 19659	*Bacillus* spp.	1.29×10^8<br>6.10×10^8<br>3.60×10^8	9/9 (100%)	3.57×10^5<br>4.97×10^5<br>2.90×10^5	9/9 (100%)
Enterococcus faecium ATCC 700221	*Enterococcus faecium* | *vanA*	2.37×10^8<br>3.01×10^8<br>2.24×10^8	9/9 (100%)	9.30×10^8<br>7.80×10^8<br>9.30×10^8	12/12 (100%)[a]
Enterococcus faecalis ATCC 51575	*Enterococcus faecalis* | *vanB*	6.87×10^8<br>9.27×10^8<br>9.83×10^8	9/9 (100%)	2.90×10^9<br>2.80×10^9<br>2.57×10^9	9/9 (100%)
Listeria monocytogenes ATCC 15313	*Listeria* spp.	7.57×10^8<br>9.63×10^8<br>7.73×10^8	9/9 (100%)	6.40×10^8<br>4.33×10^8<br>8.17×10^8	9/9 (100%)
Staphylococcus aureus ATCC BAA-2312	*Staphylococcus* spp.| *Staphylococcus aureus* | *mecA/mecC*	1.52×10^7<br>2.34×10^7<br>1.88×10^7	9/9 (100%)	3.80×10^8<br>4.10×10^8<br>1.30×10^8	9/9 (100%)
Staphylococcus epidermidis ATCC 35984	*Staphylococcus* spp. |*Staphylococcus epidermidis* | *mecA/mecC*	1.55×10^8<br>4.40×10^8<br>2.84×10^8	9/9 (100%)	1.40×10^9<br>1.48×10^9<br>1.52×10^9	9/9 (100%)
Staphylococcus lugdunensis ATCC 49576	*Staphylococcus* spp.| *Staphylococcus lugdunensis*	5.67×10^8<br>7.43×10^8<br>8.90×10^8	9/9 (100%)	2.83×10^8<br>2.92×10^8<br>2.65×10^8	9/9 (100%)
Streptococcus agalactiae ATCC 12386	*Streptococcus* spp.| *Streptococcus agalactiae*	1.21×10^9<br>1.32×10^9<br>1.12×10^9	9/9 (100%)	1.69×10^9<br>1.48×10^9<br>2.03×10^9	9/9 (100%)
Streptococcus anginosus ATCC 33397	*Streptococcus* spp.| *Streptococcus anginosus* group	7.57×10^8<br>4.97×10^8<br>3.63×10^8	9/9 (100%)	3.30×10^6<br>6.13×10^6<br>1.90×10^7	9/9 (100%)
Streptococcus pneumoniae ATCC 49619	*Streptococcus* spp.| *Streptococcus pneumoniae*	8.30×10^8<br>8.40×10^8<br>9.80×10^8	9/9 (100%)	1.35×10^7<br>1.92×10^7<br>1.54×10^8	9/9 (100%)
Streptococcus pyogenes ATCC 700294	*Streptococcus* spp.| *Streptococcus pyogenes*	3.43×10^8<br>3.40×10^8<br>4.70×10^8	9/9 (100%)	7.60×10^8<br>5.00×10^8<br>4.10×10^8	9/9 (100%)
Negative Blood	None	Ring Negative	0/3 (0%)		

[a]False Positive *Bacillus* spp. result in one replicate. Three additional replicates were tested and no additional FPs were observed.

Table S8: Inclusivity study of LIAISON PLEX® Gram-Positive Blood Culture Assay

Reportable Target (Genus)	Reportable Target (Species)	Organism	# of strains	% Detected
Bacillus spp.	N/A	*Bacillus cereus*	4	100%
		Bacillus licheniformis	2	100%
		Bacillus subtilis	3	100%
		Bacillus thuringiensis	2	100%
N/A	*Enterococcus faecalis*	*Enterococcus faecalis*	9	100%
	Enterococcus faecium	*Enterococcus faecium*	9	100% [a]
Listeria spp.	N/A	*Listeria grayi*	2	100%
		Listeria innocua	2	100%
		Listeria ivanovii	2	100%
		Listeria monocytogenes	6	100%
		Listeria seeligeri	2	100%
		Listeria welshimeri	2	100%
Staphylococcus spp.	*S. aureus*	*Staphylococcus aureus*	43	100%
	S. epidermidis	*Staphylococcus epidermidis*	8	100%
	S. lugdunensis	*Staphylococcus lugdunensis*	5	100%
	N/A	*Staphylococcus argenteus*	2	100% [b]
		Staphylococcus auricularis	2	100%
		Staphylococcus capitis	2	100%
		Staphylococcus caprae	1	100%
		Staphylococcus cohnii	2	100%
		Staphylococcus haemolyticus	2	100%
		Staphylococcus hominis	3	100%
		Staphylococcus intermedius	2	100%
		Staphylococcus muscae	1	100% [c]
		Staphylococcus pasteuri	1	100%
		Staphylococcus saccharolyticus	3	100%
		Staphylococcus saprophyticus	2	100%
		Staphylococcus schleiferi	1	100%
		Mammaliicoccus (Staphylococcus) sciuri	2	100%
		Staphylococcus simulans	2	100%
		Staphylococcus warneri	2	100%
		Staphylococcus xylosus	1	100%
Streptococcus spp.	*S. agalactiae*	*Streptococcus agalactiae*	5	100%
	S. anginosus Group	*Streptococcus anginosus*	2	100%
		Streptococcus constellatus	3	100%
		Streptococcus intermedius	2	100%
	S. pneumoniae	*Streptococcus pneumoniae*	5	100%
	S. pyogenes	*Streptococcus pyogenes*	5	100%
	N/A	*Streptococcus bovis*	3	100%
		Streptococcus dysgalactiae	2	100%
		Streptococcus equi	2	100%
		Streptococcus equinus	2	100%
		Streptococcus gallolyticus	3	100%
		Streptococcus gordonii	2	100%
		Streptococcus infantarius subsp. *coli*	1	100%
		Streptococcus infantis	2	100%

	Streptococcus mitis	2	100%
	Streptococcus mutans	2	88.9% [d]
	Streptococcus oralis	2	100%
	Streptococcus parasanguinis	2	100%
	Streptococcus peroris	1	100%
	Streptococcus pseudopneumoniae	1	100%
	Streptococcus salivarius	1	100%
	Streptococcus sanguinis	2	100%

[a]One replicate from one strain resulted in a false positive (FP) *S. lugdunensis* result, three additional replicates were run for that strain. No additional FPs were observed.
[b]*Staphylococcus argenteus* cross-reacts with *S. aureus*.
[c]*Staphylococcus muscae* cross-reacts with *Listeria* spp.
[d]One strain of *Streptococcus mutans,* ATCC 25175*,* was not detected as *Streptococcus* spp. in one of three initial replicates tested. Three additional replicates were run, giving an overall detection rate for *Streptococcus* spp. of 5/6 for that strain*. S. mutans* strain 31383 was fully detected in all replicates.

Table S9: Interfering substances study of LIAISON PLEX® Gram-Positive Blood Culture Assay

	Organism	Interfering Substance & Tested Concentration						
		Unconjugated Bilirubin 20 mg/dL	Conjugated Bilirubin 20 mg/dL	Hemoglobin 14 g/L	Intralipid 3 g/dL	γ-globulin 6 g/dL	Sodium polyanethol-sulfonate 0.25% w/v	No interferent
% Positive Calls	*Staphylococcus aureus*	100%	100%	100%	100%	100%	100%	100%
	Staphylococcus epidermidis	100%	100%	100%	100%	100%	100%	100%
	Streptococcus pneumoniae	100%	100%	100%	100%	100%	100%	100%
	Streptococcus agalactiae	100%	100%	100%	100%	100%	100%	100%
	Enterococcus faecalis	100%	100%	100%	100%	100%	100%	100%
	Enterococcus faecium	100%	100%	100%	100%	100%	100%	100%
	Negative Blood Matrix	0%	0%	0%	0%	0%	0%	0%

Table S10: Range of CFU/mL by organism across all tested bottle types

Gram-Positive Organisms	Strain ID	Expected Result	Bottle Titer Range (CFU/mL)
Bacillus cereus	ATCC 10702	*Bacillus* spp.	6.10×10^{7a}–4.40×10^{8}
Bacillus subtilis	ATCC 19659	*Bacillus* spp.	4.90×10^{5}–3.73×10^{8}
Enterococcus faecalis	ATCC 51575	*Enterococcus faecalis* | *vanB*	8.63×10^{7}–3.50×10^{9b}
Enterococcus faecalis	Clinical Isolate CLCS VRE-1	*Enterococcus faecalis* | *vanA*	9.20×10^{6}–6.50×10^{8}
Enterococcus faecium	ATCC 700221	*Enterococcus faecium* | *vanA*	3.17×10^{7}–9.77×10^{8}
Enterococcus faecium	ATCC 51858	*Enterococcus faecium* | *vanB*	3.83×10^{6}–5.50×10^{8}
Listeria ivanovii	ATCC 700402	*Listeria* spp.	1.50×10^{8}–1.40×10^{9}
Listeria monocytogenes	ATCC 15313	*Listeria* spp.	6.60×10^{7}–1.88×10^{9}
Staphylococcus aureus	ATCC BAA-2312	*Staphylococcus* spp. | *S. aureus* | *mecA*/mec*C*	8.00×10^{6}–1.29×10^{9}
Staphylococcus aureus	CDC AR-0227	*Staphylococcus* spp. |*S. aureus* | *mecA*/*C*	1.43×10^{7}–3.50×10^{9}
Staphylococcus epidermidis	ATCC 35984	*Staphylococcus* spp. | *S. epidermidis* |*mecA*/mec*C*	6.93×10^{6}–1.43×10^{9}
Staphylococcus lugdunensis	ATCC 49576	*Staphylococcus* spp. | *S. lugdunensis*	2.20×10^{7}–3.46×10^{9}
Streptococcus agalactiae	ATCC 12386	*Streptococcus* spp. | *S. agalactiae*	2.70×10^{7}–1.71×10^{9}
Streptococcus anginosus	ATCC 33397	*Streptococcus* spp. | *S. anginosus* Group	1.82×10^{7}–4.37×10^{9}
Streptococcus constellatus	ATCC 27823	*Streptococcus* spp. | *S. anginosus* Group	4.00×10^{7}–2.53×10^{9}
Streptococcus pneumoniae	ATCC 49619	*Streptococcus* spp. | *S. pneumoniae*	2.70×10^{6}–2.88×10^{9}
Streptococcus pyogenes	ATCC 700294	*Streptococcus* spp. | *S. pyogenes*	2.80×10^{6}–2.07×10^{9}
Corynebacterium diphtheriae	ATCC 27010	No target detected	2.80×10^{6}–1.31×10^{9}
Corynebacterium striatum	ATCC 43735	No target detected	4.30×10^{7}–1.17×10^{9}
Cutibacterium acnes	ATCC 6919	No target detected	2.83×10^{7}–5.90×10^{9}

[a]No viable counts were achieved for *B. cereus* in two of the BD BACTEC™ Standard Anaerobic media bottles.
[b]Two bottles tested at initial bottle positivity with titers of 8.63×10^{7} and 1.46×10^{8} CFU/mL were not fully detected. Bottles grown to initial bottle positivity +8 hours were used to confirm that the results were not due to bottle matrix.

Table S11: LIAISON PLEX® Gram-Positive Blood Culture Assay on-panel competitive inhibition summary

On-Panel High Titer Target	Positivity	On-Panel Low Titer Target	Concentration (CFU/mL)	Positivity
Enterococcus faecalis 1.54×10^9 CFU/mL	100%	*Staphylococcus epidermidis*	1.76×10^8	100%
	100%	*Enterococcus faecium*	8.96×10^7	100%
Enterococcus faecium 4.68×10^8 CFU/mL	100%	*Staphylococcus epidermidis*	1.76×10^8	100%
	100%	*Enterococcus faecalis*	3.71×10^8	100%
Staphylococcus aureus 2.28×10^8 CFU/mL	100%	*Staphylococcus epidermidis*	1.76×10^8	100%
	100%	*Streptococcus agalactiae*	4.48×10^8	100%
Staphylococcus epidermidis 8.88×10^8 CFU/mL	100%	*Enterococcus faecalis*	3.71×10^8	100%
	100%	*Enterococcus faecium*	8.96×10^8	100%
	100%	*Staphylococcus aureus*	7.52×10^6	100%
	100%	*Staphylococcus lugdunensis*	2.27×10^8	100%
	100%	*Streptococcus pneumoniae*	3.92×10^8	100%
Staphylococcus lugdunensis 1.59×10^8 CFU/mL	100%	*Staphylococcus epidermidis*	1.76×10^8	100%
Streptococcus agalactiae 9.54×10^8 CFU/mL	100%	*Staphylococcus aureus*	7.52×10^6	100%
Streptococcus pneumoniae 8.10×10^6 CFU/mL	100%	*Staphylococcus epidermidis*	1.76×10^8	100%

Table S12: LIAISON PLEX Gram-Positive Blood Culture Assay microbial interference summary

On-Panel Low Titer Target	Positivity	Off-Panel High Titer Target	Concentration (CFU/mL)	Positivity
Staphylococcus epidermidis 1.76×10^8 CFU/mL	100%	*Escherichia coli*	1.12×10^9	0%
	100%	*Klebsiella pneumoniae*	8.58×10^8	0%
	100%	*Proteus mirabilis*	2.72×10^7	0%
Enterococcus faecium 8.96×10^7 CFU/mL	100%	*Escherichia coli*	1.12×10^9	0%
	100%	*Klebsiella pneumoniae*	8.58×10^8	0%
	100%	*Proteus mirabilis*	2.72×10^7	0%
N/A	N/A	*Escherichia coli*	1.12×10^9	0%
	N/A	*Klebsiella pneumoniae*	8.58×10^8	0%
	N/A	*Proteus mirabilis*	2.72×10^7	0%
